# Snake Venom Protease Detection and Inhibition in Serum

**DOI:** 10.1002/cmdc.202501099

**Published:** 2026-03-31

**Authors:** Mareike Riedel, Christian Kersten

**Affiliations:** ^1^ Institute of Pharmaceutical and Biomedical Science Johannes Gutenberg University Mainz Germany; ^2^ Institute for Quantitative and Computational Biosciences Johannes Gutenberg University Mainz Germany

**Keywords:** envenoming diagnostics, fluorescence‐based assay, metalloproteases, serine proteases, small molecule inhibitors, snake venom proteases, viper venom

## Abstract

Snake bites remain major threats with only limited diagnostic and therapeutic options. Current antibody‐based antivenoms show only limited effectiveness against local tissue damage and require complex manufacturing and cold chain logistics. To overcome these limitations, a fluorescence‐based assay was established to enable the sensitive detection of snake venom metalloprotease (SVMP) and serine protease (SVSP) activities in crude viper venoms from *Crotalus atrox*, *Bothrops jararaca,* and *Echis carinatus*. The assays showed species‐specific activity profiles with detection limits in the sub‐microgram per 45 and 100 µL range for SVSP and SVMP, respectively. Notably, protease activity in bovine and human blood sera, with only a slight loss of sensitivity compared to isolated venom in buffer, can be measured. Inhibitory effects of small molecule protease inhibitors, batimastat, marimastat, ilomastat, nafamostat, and leupeptin, were determined, showing strong SVMP or SVSP inhibition, respectively. Based on this proof‐of‐concept, the combination of such activity‐based assays with selective small‐molecule inhibitors could open new opportunities for rapid venom detection in diagnosis and complementary therapy, particularly in resource‐poor settings.

## Graphical abstract

A fluorescence assay enables sensitive detection of the activities of snake venom metalloproteases (SVMP) and serine proteases (SVSP) from viper venoms in buffer and serum. In combination with effective low‐molecular‐weight inhibitors such as ilomastat and nafamostat, this approach offers new prospects for the rapid diagnosis and adjunctive therapy of snake bites.

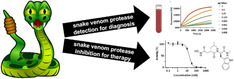

## 1 Introduction

**FIGURE 1:**
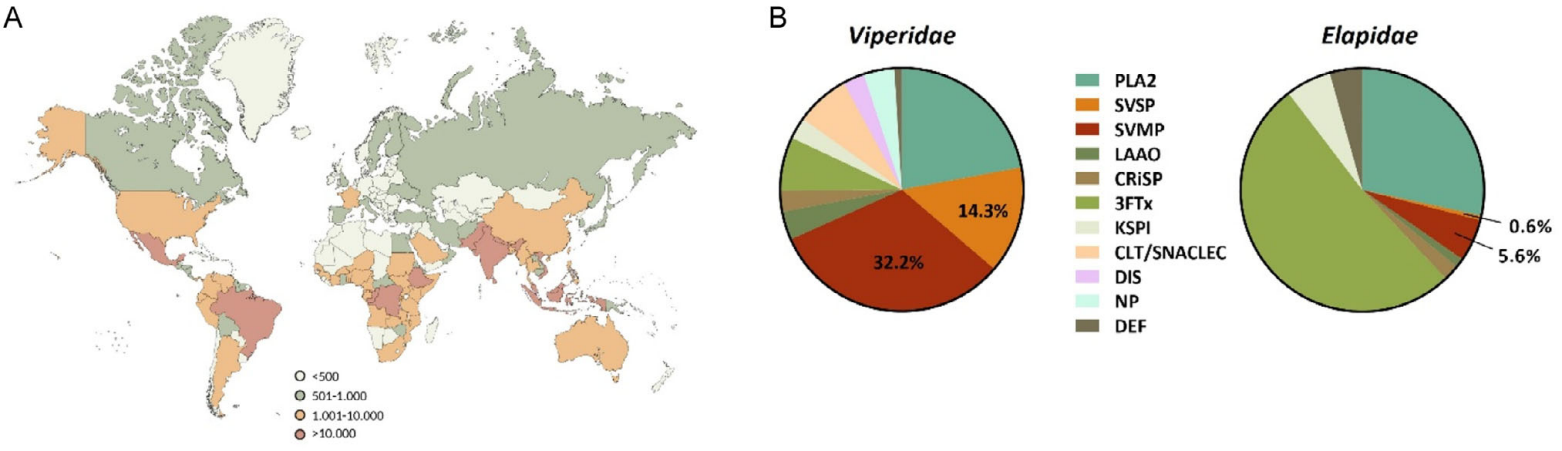
(A) World map showing the estimated total number of snake bites per country per year, based on reported cases (lower estimate) [2]. (B) Composition of *Elapidae* and *Viperidae* snake venoms. PLA2, phospholipase A2; SVMP, snake venom metalloprotease; SVSP, snake venom serine protease; LAAO, l‐amino acid oxidase; CRiSP, cysteine‐rich secretory protein; 3FTx, three‐finger toxin; KSPI, Kunitz‐type serine protease inhibitor; CTL/SNACLEC, C‐type lectin and C‐type lectin‐like protein; DIS, disintegrin; DEF, defensin; NP, natriuretic peptide.

Snakebites, classified as neglected tropical diseases, pose a major global health threat. Annually, around 5.4 million bites are reported, leading to 0.5–2.7 million cases of envenoming [1, 2] (Figure 1A). Approximately 81,000 to 138,000 people die from complications, and over 400,000 suffer severe and permanent damage [1].

The composition of snake venoms has been studied through proteomic analyses (Figure 1B) [3]. The investigation of venoms from over 200 snake species in the *Elapidae* and *Viperidae* families revealed significant inter‐ and intraspecific variation [4, 5, 6, 7]. In *Elapidae*, secreted phospholipases A_2_ (sPLA_2_) and three‐finger toxins (3FTx) are predominant, typically comprising about 80% of the venom proteome, though their proportions vary widely between species [3]. In contrast, *Viperidae* venoms mainly consist of sPLA_2_ [8, 9, 10], snake venom metalloproteases (SVMPs) [9, 10, 11, 12], and snake venom serine proteases (SVSPs) [9, 10, 13, 14], which together account for about 70% of venom composition [3]. These enzymes target various components of the hemostatic system, affecting coagulation [15, 16], fibrinolysis, platelet aggregation [17], and the kallikrein‐kinin system, thereby causing complex circulatory disturbances [9, 13, 14, 18].

SVSPs are an important family of toxins found in snake venom and are present in almost all vipers [19]. They belong to the trypsin‐like family of serine proteases [20]. Their effect is primarily directed at the hemostatic system and leads to edema formation [21], blood clotting disorders [20], increased fibrinolysis [22] and platelet aggregation [21]. SVSPs are structurally closely related to trypsin, chymotrypsin and thrombin [21].

SVMPs are another major group of bioactive components in many *Viperidae* venoms. *Viperidae* venom contains between 10% to more than 65% SVMPs [3, 23]. These multidomain proteins induce hemorrhage, degrade fibrin(ogen), inhibit platelet aggregation, and trigger pro‐inflammatory and pro‐apoptotic responses. Their hemorrhagic effect results primarily from the degradation of capillary basement membrane components, particularly type IV collagen, compromising vascular integrity [11, 12]. The mechanism of these effects is not fully understood so far [24]. SVMPs are classified into PI, P‐II, and P‐III groups based on increasing structural complexity. PI SVMPs contain only a catalytic metalloprotease domain, while P‐II and P‐III include additional disintegrin or disintegrin‐like and cysteine‐rich domains, respectively [25, 26].

Currently, only polyclonal antivenoms produced from animals are available for treatment [27, 28, 29, 30]. They are effective in cases of systemic envenomation, but less so in cases of local tissue damage, and can trigger allergic reactions [31, 32, 33, 34]. Availability, cost, cold chain requirements and regional supply structures limit their global use. In addition, ‘dry bites’ (10%–50%) complicate clinical assessment and often lead to unnecessary administration of antivenom [35, 36]. In contrast, delayed antivenom therapy can have serious consequences. Diagnosis is usually made clinically based on the progression of symptoms. A simple procedure is the 20‐min Whole Blood Clotting Test (WBCT20), which, however, only detects coagulopathy and is unreliable in the early stages [37]. Rapid tests such as ELISAs or lateral flow assays are under development; some are available regionally (e.g., Australia), but most are species‐specific and have not yet been widely validated clinically [38, 39, 40]. Molecular methods (polymerase chain reaction or mass spectrometry) offer high specificity but are currently too complex for routine use [41, 42, 43]. Overall, there is a lack of robust, rapid and widely applicable diagnostic methods for reliably distinguishing between bites from non‐venomous snakes, dry bites and mild and severe poisoning before symptoms appear.

The limitations of conventional antivenoms and diagnostic procedures highlight the need for new approaches. Recombinant and humanized antibodies improve safety and, can be effective against multiple toxins in snake venom [44]. Nanobodies are characterized by high stability and have great therapeutic potential [45]. Low‐molecular‐weight inhibitors of PLA_2_, SVMPs and SVSPs are promising, particularly due to their oral availability and suitability for resource‐poor regions; the PLA_2_ inhibitor methylvarespladib is currently in clinical phase 2 trials [46, 47, 48]. The combined further development of therapeutic and diagnostic approaches thus represents a key step towards improving snake bite treatment.

We investigated the venoms of three *Viperidae* species to characterize proteolytic SVMP and SVSP activities using a fluorescence‐based assay. In addition, selected low‐molecular‐weight inhibitors were tested for their broad‐spectrum inhibitory activity. Complementing this, a serum test system for the detection of venom components was developed, which provides a proof‐of‐concept basis for future diagnostic applications.

## 2 Results and Discussion

**FIGURE 2:**
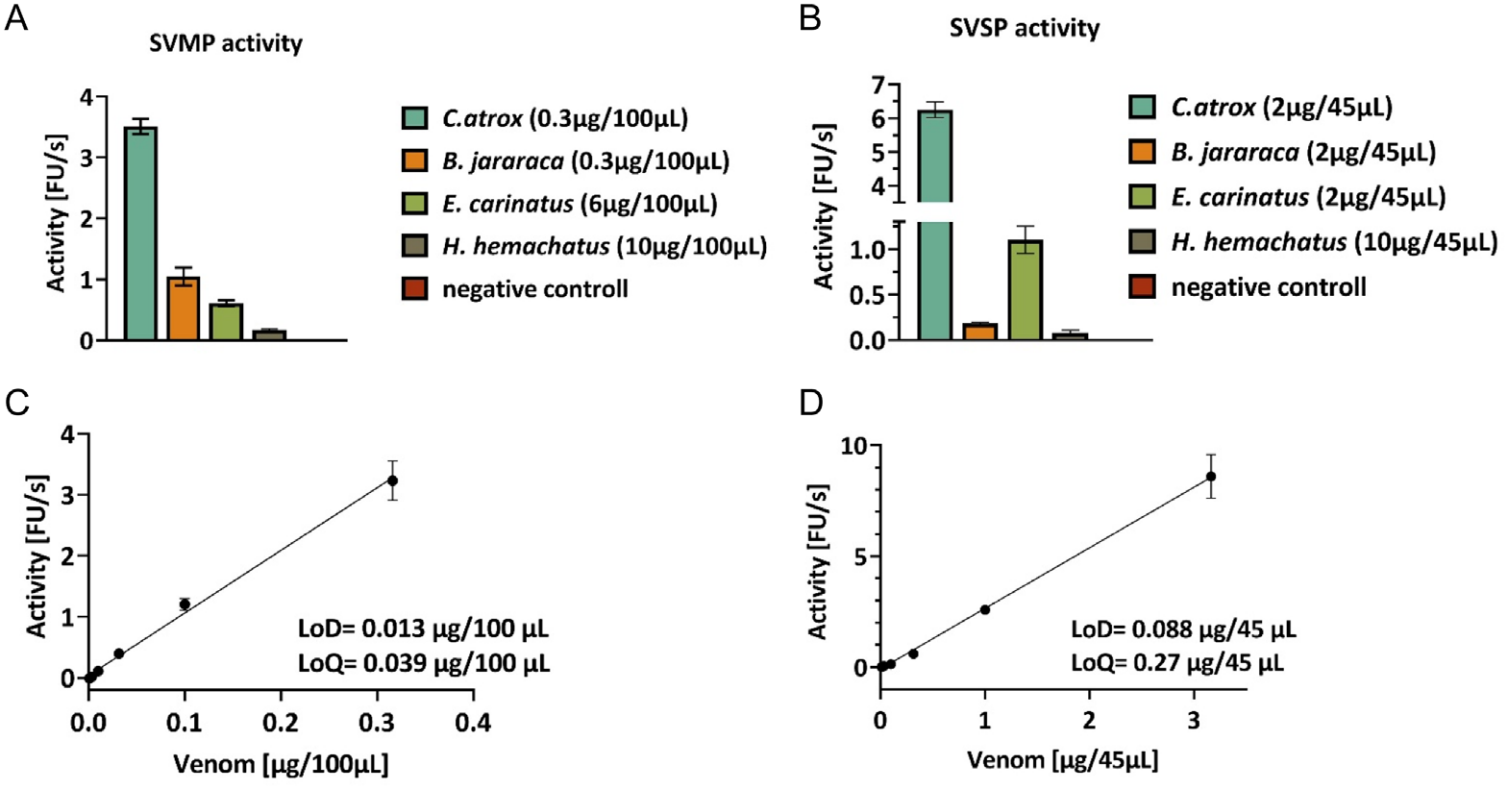
(A) SVMP activity over 10 min at room temperature for four different venoms. (B) SVSP activity of four venoms over 10 min. (C) Determination of LoD and LoQ for the SVMP assay setup for *C. atrox* venom. (D) LoD and LoQ determinations for SVSP assay for *C. atrox* venom.

To investigate SVSP and SVMP activity and inhibition, viper venoms of *Crotalus atrox*, *Bothrops jararaca,* and *Echis carinatus* were tested in a fluorescence‐based assay. Due to the high sequence similarity within different SVSPs and to human thrombin‐like serine proteases and SVMPs to human metalloproteases (MMPs), similar structures, and substrate preferences were assumed. Peptide **A**, a variant of a reported FRET‐substrate for human MMP9 (Abz‐Glu‐Pro‐Leu‐Gly‐(4‐I‐Phe)‐Ala‐Ser‐Arg‐Lys(Dnp)‐amidation), was used to detect SVMP activity (Figure 2A) [49]. The analysis showed that the venom of *C. atrox* exhibited the highest activity with this substrate, followed by *B. jararaca*. In the case of *E. carinatus*, the amount of venom had to be increased to achieve stable activity. These results confirm that at least some of the often multiple SVMPs of viper venoms (Table S1) can be detected using the selected substrate. For comparison, the venom of the elapid, *Hemachatus hemachatus*, was also tested. Here, very low SVMP activity could be detected, which is probably due to the significantly lower proportion of SVMPs in elapid venoms compared to viper venoms (Figure 1B) [3]. These observations are consistent with proteomic data showing the highest proportion of SVMPs for *C. atrox*. Differences in substrate affinity could also arise from structural variations in the active centers of the metalloproteases and not only their abundance. Further, the SVSP activity in the four different snake venoms was tested using a known trypsin substrate (Boc‐Leu‐Arg‐Arg‐AMC, Figure 2B). Here the activity also correlated with the reported amount of SVSP in each venom [3]. *C. atrox* again showed the highest activity, whereas the venom of *H. hemachatus* showed no proteolytic activity in this assay setup.

In order to evaluate the analytical sensitivity of the fluorescence assays, limit of detection (LoD) and limit of quantification (LoQ) values were also determined for SVMP and SVSP activity in buffer (Table 1, Figure 2C,D and Figure S1). For the SVMP assay, *C. atrox* showed the lowest detection and quantification limits with a LoD of 0.013 µg/100 µL and a LoQ of 0.039 µg/100 µL, followed by *B. jararaca* (LoD = 0.21 µg/100 µL; LoQ = 0.64 µg/100 µL) and *E. carinatus* (LoD = 0.28 µg/100 µL; LoQ = 0.84 µg/100 µL, Figure S1). This data confirms that *C. atrox* has the highest SVMP activity under the conditions used and can be detected even in very low amounts.

**TABLE 1:** Determination of LoD and LoQ of SVMP and SVSP activity in Tris buffer.

Venom	SVMP [µg/100 µL]		SVSP [µg/45 µL]	
	LoD	LoQ	LoD	LoQ
*C. atrox*	0.013	0.039	0.088	0.27
*B. jararaca*	0.21	0.64	0.33	0.99
*E. carinatus*	0.28	0.84	0.066	0.20

A similar result was obtained in the SVSP assay. *C. atrox* showed the highest detection sensitivity with LoD = 0.088 µg/45 µL and LoQ = 0.27 µg/45 µL. *B. jararaca* was significantly higher with LoD = 0.33 µg/45 µL and LoQ = 0.99 µg/45 µL, while *E. carinatus* showed surprisingly low limits with LoD = 0.066 µg/45 µL and LoQ = 0.20 µg/45 µL, suggesting an overall weaker SVSP signal. These data show that the assays developed are suitable for mapping species‐specific differences in enzyme composition and activity, while also providing a robust basis for subsequent inhibitor studies. Additionally, kinetic parameters were determined according to the Michaelis–Menten model (Figure S2). It should be noted that these are not single enzymes, but enzyme mixtures with potentially variable composition and substrate affinity. The calculated *K*_M_ values for SVMP activity differed only slightly between the three viper venoms (*C. atrox*: *K*_M_ = 15 ± 2.9 µM; *B. jararaca*: *K*_M_ = 24 ± 5.3 µM; *E. carinatus*: *K*_M_ = 15 ± 2.7 µM). However, the maximum turnover rates showed bigger differences as seen in the initial activity assessment (*C. atrox*: *v*_max_ = 5.1 ± 0.5 FU/s; *B. jararaca*: *v*_max_ = 1.8 ± 0.2 FU/s; *E. carinatus*: *v*_max_ = 0.3 ± 0.02 FU/s). Similarly, for SVSPs, *C. atrox* showed the highest activity compared to the other viper venoms. The venom of *H. hemachatus* again served as an *Elapidae* reference and showed no detectable SVSP activity (Figure 2B). The *K*_M_ values determined for *C. atrox* and *B. jararaca* were in a similar range at 67 ± 5.9 and 77 ± 9.0 µM, respectively, while *E. carinatus* showed a higher value >400 µM. Interestingly, the *v*_max_ values of *C. atrox* and *E. carinatus*, at 14.6 ± 0.5 and 14.9 ± 0.5 FU/s, respectively, were significantly higher than that of *B. jararaca* (*v*_max_ = 0.23 ± 0.01 FU/s) (Figure S3)

**FIGURE 3:**
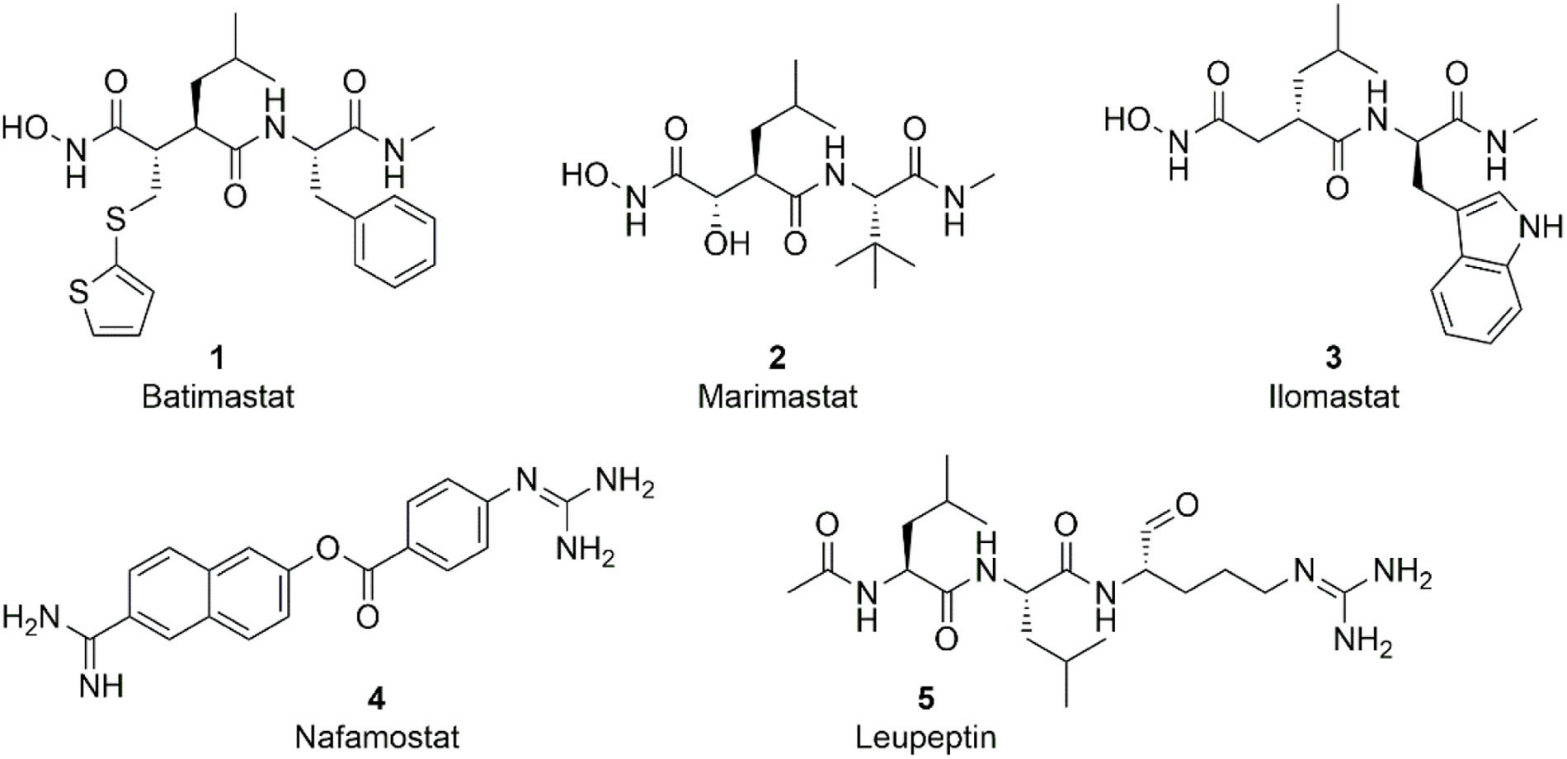
Inhibitors elucidated for SVMP inhibition (**1–3**) and SVSP inhibition (**4**, **5**).

Using these SVMP and SVSP activity assays, inhibition was investigated using reported MMP and serine protease inhibitors (Figure 3) [50, 51, 52, 53, 54]. Batimastat (**1**), marimastat (**2**), and ilomastat (**3**) were used to inhibit the SVMP component. Nafamostat (**4**) and leupeptin (**5**) were tested for SVSP inhibition.

MMP‐inhibitors **1**–**3** were originally developed in an oncological context and showed high efficacy against various human metalloproteases with published IC_50_‐values < 100 nM_._ [50, 54] In recent years, batimastat and marimastat have gained increasing attention in the context of neutralizing SVMP activities in snake venoms. Several studies on *C. atrox* and *B. jararaca* already exist that show a significant reduction in SVMP activity after the addition of these inhibitors [51]. For both species' venoms, IC_50_‐values in the low nanomolar range for batimastat and marimastat were described [55, 56, 57]. We expanded this study by the additional snake species *E. carinatus* and the metalloprotease inhibitor, ilomastat. It should be noted that the IC_50_‐values determined do not refer to a single isolated enzyme, but to the whole enzyme mixture of the venom. This can lead to a certain degree of variability, as individual SVMP isoforms within a venom can react differently to the inhibitors. Nevertheless, the IC_50_‐values obtained provide a functionally relevant assessment of the inhibitory potency against the total venom and thus allow an evaluation of the suitability of these inhibitors as potential adjuvant therapeutics in antivenom strategies. Dose‐dependent SVMP inhibition was measured for **1**–**3** with all three viper venoms (Table 2 and Figure S4).

**TABLE 2:** IC_50_ values of inhibitors 1–3 against three viper venoms’ SVMP activity.

Inhibitor	IC_50_ [nM] for SVMP activity		
	*C. atrox*	*B. jararaca*	*E. carinatus*
Batimastat	126 ± 10	38.2 ± 2.8	108 ± 13
Marimastat	17.0 ± 1.3	10.1 ± 0.9	178 ± 14
Ilomastat	9.9 ± 0.4	9.7 ± 0.7	176 ± 16

The determination of IC_50_ values confirmed significant species‐specific differences in the inhibition of SVMP activity by the tested inhibitors [47, 51]. For *C. atrox* venom, ilomastat showed the highest inhibitory effect with an IC_50_ of 9.9 ± 0.4 nM, followed by marimastat (17.0 ± 1.3 nM). Batimastat showed significantly lower inhibition of 126 ± 10 nM. In the case of *B. jararaca*, a similar activity profile was observed, with ilomastat and marimastat showing similar IC_50_‐values of 9.7 ± 0.7 and 10.1 ± 0.9 nM, respectively. Batimastat was only slightly less potent (38.2 ± 2.8 nM). *B. jararaca* SVMP activity thus shows an overall higher sensitivity to all three inhibitors evaluated. *E. carinatus* showed a different inhibition profile: The IC_50_‐values for batimastat (110 ± 13 nM) and marimastat (178 ± 14 nM) were higher than for the other two venoms. Ilomastat showed similar inhibition with IC_50_ of 176 ± 16 nM. However, it should be noted that, due to the significantly lower detectable SVMP activity, a twenty‐fold higher venom concentration had to be used in the assay for *E. carinatus* to obtain a sufficient signal.

In addition, the inhibitory effect of two serine protease inhibitors, nafamostat and leupeptin, was evaluated (Table 3 and Figure S5). For nafamostat, the effect against the SVSP activity of different *Bothrops* species showed only low inhibition at a concentration of 10 µM for *B. jararaca* in a previous study [55]. In line with these findings, nafamostat showed higher inhibition of SVSP in *C: atrox* and *E. carinatus* with IC_50_‐values of 36 ± 2 and 760 ± 70 nM, respectively, compared to *B. jararaca* SVSP with an IC_50_‐value higher than 10 µM. Leupeptin had IC_50_‐values in the one‐digit micromolar range for *C. atrox* (1.6 ± 0.1 µM) and *B. jararaca* (5.1 ± 0.8 µM), whereas it is 17 ± 1 µM for *E. carinatus* venom SVSP inhibition.

**TABLE 3:** IC_50_ values of inhibitors 4 and 5 against three viper venoms’ SVSP activity.

**Inhibitor**	IC_50_ [µM] for SVSP activity		
	***C. atrox***	***B. jararaca***	***E. carinatus***
Nafamostat	0.036 ± 0.002	> 10	0.76 ± 0.07
Leupeptin	1.6 ± 0.1	5.1 ± 0.8	17 ± 1

One challenge of snake bites beyond therapeutic neutralization of toxins is its time‐critical diagnosis. In many regions with high incidence, there is a lack of standardized procedures for the rapid functional determination of enzymatic activity in patient material. Therefore, the choice of antivenom is often empirical and thus associated with an increased risk of incorrect treatment and unintended side effects. Therefore, the assay was tested for its suitability to detect SVSP and SVMP activity in biological samples. By adding defined concentrations of the three viper venoms investigated to bovine and human sera and measuring proteolytic activity, it was shown that both SVMP and SVSP activity can be measured reproducibly in this type of biological sample. The LoD and LoQ values for SVMP activity were determined in bovine and human sera (Table 4 and Figure S6). The measurement was performed with peptide **B** (Mca‐Glu‐Pro‐Leu‐Gly‐(4‐I‐Phe)‐Ala‐Ser‐Arg‐Lys(Dnp)‐amidation). The FRET donor/acceptor pair was changed compared to peptide **A** to improve the signal‐to‐noise ratio against serum background. For *C. atrox* venom, the LoD was 0.051 µg/100 µL and the LoQ was 0.15 µg/100 µL, while for *B. jararaca*, values of 0.17 µg/100 µL (LoD) and 0.52 µg/100 µL (LoQ) were detected in bovine serum. *E. carinatus* venom showed higher thresholds at 1.0 µg/100 µL (LoD) and 3.0 µg/100 µL (LoQ) (Table 4 and Figures S6B–D) in bovine serum. While *C. atrox* SVMP activity was already quantifiable at 0.039 µg/100 µL in buffer, the LoQ in bovine serum is 0.15 µg/100 µL. In the case of *B. jararaca*, however, the LoQ values in bovine serum (0.52 µg/100 µL) and in buffer (0.64 µg/100 µL) are more similar, indicating that the activity of this venom is relatively robust against matrix effects and that the assay sensitivity is largely maintained. In the case of *E. carinatus*, however, the LoQ change is larger (LoQ buffer: 0.84 µg/100 µL, LoQ bovine serum: 3.0 µg/100 µL), which is consistent with the lower basal enzyme activity and the need for higher venom concentrations. In addition, the LoD and LoQ values were also determined in human serum to increase the transferability to reality as a diagnostic tool for snake envenoming in humans (Figure S6E–G). Here the LoD and LoQ values for *C. atrox* (LoQ of 0.44 µg/100 µL) and *E. carinatus* (LoQ of 7.0 µg/100 µL) venoms are slightly increased in human serum compared to bovine. For *B. jararaca*, the LoD and LoQ were similar in bovine (LoQ of 0.52 µg/100 µL) and human sera (LoQ of 0.38 µg/100 µL). Summarized, the LoD and LoQ determination of the SVMP activity was possible for all venoms in buffer, bovine and human sera. *E. carinatus* has in all media the highest LoD and LoQ for SVMP activity. For SVSP activity, the LoD and LoQ determination in both sera were also investigated (Table 4 and Figure S7). A direct comparison with LoD and LoQ determined in the buffer shows that the LoQ in sera is higher, as we also observed for SVMP activity. Likewise, LoD and LoQ increased from bovine to human serum. The increase of LoD and LoQ in the serum measurement can be explained by the interference of serum proteins such as metalloproteases or trypsin‐like serine proteases from the coagulation cascade with the substrates used. In addition, the substrate used for SVSP activity is a substrate for the detection of trypsin‐like protease activity, likely causing higher background signals. Overall, the results from the buffer experiments could be confirmed for both the bovine and the human sera experiments with in general slightly higher values of LoD and LoQ in the sera.

**TABLE 4:** LoD and LoQ determination of three venoms’ SVMP and SVSP activity in bovine and human blood serum.

	Bovine serum				human serum			
	SVMP [µg/100 µL]		SVSP [µg/45 µL]		SVMP [µg/100 µL]		SVSP [µg/45 µL]	
Venom	LoD	LoQ	LoD	LoQ	LoD	LoQ	LoD	LoQ
*C. atrox*	0.051	0.15	0.17	0.52	0.14	0.44	0.88	2.7
*B. jararaca*	0.17	0.52	7.0	21	0.13	0.38	9.8	30
*E. carinatus*	1.0	3.0	1.4	4.1	2.3	7.0	1.6	4.8

To evaluate the physiological relevance of the determined detection limits, venom concentrations under realistic envenoming conditions were estimated. Literature data for *C. atrox* showed a high degree of variability in the amount of venom injected. In a study using electrical venom extraction, an amount of 178–354 mg of venom (dry weight) per bite was determined over a period of several weeks [58]. Pharmacokinetic studies on snake venoms showed that systemic bioavailability is influenced by numerous factors, including the composition of the venom and its distribution volume, the anatomical location of the bite, *i.v.*, *i.m.* or *s.c.* injection, and other individual factors. Accordingly, the literature describes venom bioavailability in the range of ≈ 4–80% [59]. For viper venoms, blood concentrations in the range of 10–100 µg/L early after the bite were described [59]. In individual cases, this might be higher, reaching up to 1000 µg/L as well as closer to the bite mark. Considering that the SVMP assay enables reliable quantification between 0.38–6.9 µg per 100 µL in human serum samples, the achieved LoQ‐values are higher than the physiologically relevant range. Similar results were also found in the investigation of SVSP activity in serum. Here, the detectable amount of snake venom was 0.88–7.0 µg per 45 µL in human serum (Table 4). These findings indicate that the proof‐of‐concept to detect SVMP and SVSP activity can work for diagnosis of viper bites from biological samples like serum, but the assay possesses insufficient analytical sensitivity to detect enzymatic activity at toxin concentrations expected in envenomed patients. Therefore, this assay demonstrates SVMP and SVSP enzyme activity measurements, even in the complex composition of serum samples, as a potential snake bite envenoming detection technique. In particular, the robust measurement of snake venom proteases could offer advantages over currently available measurement methods. For the low venom bioavailability, however, higher sensitivity is required to accurately detect low amounts of venom. Additionally, in the current setup, the assay is only compatible with sera, but not whole blood samples, due to fluorescence interference. Therefore, it provides a promising foundation for the future development of activity‐based diagnostic methods, like integration into (more sensitive and whole‐blood compatible) biosensor systems [60, 61], clinical laboratory tests, or potentially mobile point‐of‐care devices for immediate field application.

In addition, marimastat and nafamostat were tested in bovine and human sera (Table 5). The IC_50_‐value of marimastat was 17 nM for *C. atrox* in buffer (Table 2). In bovine serum, the inhibitory effect of marimastat was similar with an IC_50_‐value of 12 nM. Due to a higher LoQ in human serum, the amount of venom was increased from 0.3 µg/100 µL venom to 1 µg/100 µL in the assay. Therefore, the IC_50_‐value is slightly increased in human serum to 42 nM. This shows that inhibition of SVMP activity in serum is possible to a comparable extent as under buffer conditions. For the determination of marimastat's inhibitory effect against SVMP activity of *B. jararaca* in both sera, the venom amount was increased to 1 µg/100 µL. This led to an IC_50_‐value of 9.2 nM in bovine serum and an IC_50_ value of 13.0 nM in human serum, which is in line with the IC_50_ of 10.1 nM in buffer. For the *E. carinatus* venom, the LoQ of the SVMP assay in sera was too high to determine an IC_50_‐value of marimastat both in bovine and in human serum. Further, the IC_50_‐values of nafamostat for SVSP inhibition were determined in bovine and human sera, For *C. atrox* venom, the conditions in bovine serum were the same as in buffer. The IC_50_‐value of nafamoastat was slightly increased from 36 nM in buffer to 120 nM in bovine serum, maybe due to the interactions of the serine protease inhibitor with endogenous serine proteases in serum. For the IC_50_ determination in human serum, the amount of venom was increased from 2 µg/45 µL to 6 µg/45 µL, because of the higher LoQ values. A slightly higher IC_50_ of 420 nM in human serum was found, but still in the same order of magnitude as in bovine serum. For *B. jararaca* venom, the determination of the inhibitory effect of SVSP activity was not possible, because of the lower activity of this venom in general, which leads to high LoQs in sera. For *E. carinatus*, the concentration of venom was increased in both sera to 6 µg/45 µL. The IC_50_‐value of nafamostat was 750 nM in bovine serum, which is in the same range as in buffer (760 nM). In human serum, the IC_50_‐value was 1.5 µM, which is slightly increased compared to bovine.

**TABLE 5:** SVMP and SVSP inhibition assay IC_50_‐values of inhibitors 2 and 4 for three viper venoms in bovine and human bloodserum.

Inhibitor	IC_50_ [nM] in bovine serum		IC_50_ [nM] in human serum	
	Marimastat (SVMP)	Nafamostat (SVSP)	Marimastat (SVMP)	Nafamostat (SVSP)
*C. atrox*	12 ± 2.3	120 ± 41	42 ± 12	420 ± 130
*B. jararaca*	9.2 ± 3.2	n.d.	13 ± 4.4	n.d.
*E. carinatus*	n.d.	750 ± 120	n.d	1500 ± 760

n.d.: not determined due to high LoQ.

## 3 Conclusion

In this study, a fluorescence‐based assay was established for the sensitive detection and quantitative analysis of snake venom SVMP and SVSP activity. Three viper venoms were characterized, and species‐specific differences in enzyme activity were shown. The investigation of small molecule inhibitors **1**–**5** showed that compounds such as ilomastat and nafamostat effectively inhibit SVMP and SVSP activity, respectively. Therefore, they can be considered as adjuvant therapeutics alongside conventional antivenoms against viper bites, potentially in combination with PLA2 inhibitors like methylvarespladib [47]. Compared to antibody‐based therapies, following‐up such small molecule therapeutics holds the advantage of being less complex and cheaper in manufacturing, and more stable against environmental conditions like high temperature and humidity – both relevant aspects in regions of high snake bite incidence (Figure 1A) [62]. The presented assay concept can be reliably applied in complex biological samples such as blood serum, but higher sensitivity is required. These results form the proof‐of‐concept basis for the development of activity‐based diagnostic tools and open opportunities for rapid, functional detection systems for viper venoms. In combination with selective inhibitors, such technologies could significantly improve the diagnosis and treatment of snake bite envenoming – especially in resource‐limited regions where rapid, environmentally stable, and cost‐effective diagnosis and treatment is crucial.

## 4 Material and Methods

### 4.1 Venoms

Snake venoms of *C. atrox*, *B. jararaca*, *E. carinatus,* and *H. hemachatus* were commercially available as lyophilized powder from Sigma Aldrich/Merck (Merck KGaA, Darmstadt, Germany) and stored at −20 °C. Venoms were diluted in PBS buffer (pH = 7.4) as 6 or 1 mg/mL stock solution for subsequent assay experiments. Stocks solutions were stored at 4 °C for a maximum of 1 week.

### 4.2 Substrates

The fluorogenic peptide for SVSP activity determination with sequence Boc‐LRR‐AMC was purchased from Bachem (4017087; Bachem AG, Bubendorf, Switzerland). The peptides for SVMP assays were synthesized using a solid‐phase peptide synthesis (SPPS) procedure. The following side‐chain‐protected Fmoc‐protected amino acids were used for the synthesis of substrates **A** and **B**: Lys(Dnp), Arg(Pbf), Ser(tBu) and Glu(tBu). SPPS was performed using a Biotage Initiator+ Alstra system. First, the Rink Amide AM Resin (BD629082; BLD Pharmatech Co., Limited in Shanghai, China) was swollen in dichloromethane for 60 min. This was followed by Fmoc‐deprotection by incubation with 20 % piperidine in Dimethylformamide. The respective Fmoc amino acids in Dimethylformamide (5 equivalents) were coupled, with the addition of *N*,*N*‐Diisopropylcarbodiimide and Oxyma in Dimethylformamide (5 equivalents each) and *N*,*N*‐Diisopropylethylamine (100 equivalents) in *N*‐methylpyrrolidone. The reaction was run at 75 °C for 5 min. This cycle was repeated for each amino acid according to the desired peptide sequence. After completion of the last coupling, the resin was washed with DCM. To cleave the peptide, a solution of TFA:TIPS:H_2_O (4.5:0.25:0.25) was added to the resin and incubated for 2 hours at room temperature with gentle swirling. The released linear peptide was then isolated by precipitation with N_2_‐cooled diethyl ether. The suspension was centrifuged at 4 °C for 5 min at 5000×*g*, the supernatant discarded, and the peptide pellet removed for further purification. The crude peptides were purified by semi‐preparative HPLC using a Pursuit XRs 5 C18 (30.0 x 250 mm) column with an Agilent 1290 Infinity II HPLC system consisting of a 1260 VWD detector, 1290 Prep Bin Pump and 1290 Prep FC collector. Peptides were eluted using a suitable solvent gradient (solvent A: 0.1 % HCOOH in water; solvent B: 0.1 % HCOOH in acetonitrile). The obtained pure fractions were combined and lyophilized.

The purity and identity of peptides **A** and **B** were determined by LC/MS analysis using a Waters Alliance e2695 Separation Module with an analytical Pursuit XRs C18 (4.6 × 200 mm, 5.0 μm) column coupled to a Waters Acquity QDa single quadrupole detector. Peptide **A** was separated using a mobile phase of MeCN/H_2_O + 0.1% HCOOH (gradient 10:90 → 90:10; flow rate 1.5 mL/min; *t* = 10 min). Peptide **B** was separated with a mobile phase of was MeCN/H_2_O + 0.1% HCOOH (gradient 10:90 → 100:0; flow rate 2.0 mL/min; *t* = 10 min). Mass spectra were recorded in the positive mode. The UV detection wavelength was 254 nm (Figures S9 and S10).

#### 4.2.1 Peptide A

The amino acid sequence is Abz‐Glu‐Pro‐Leu‐Gly‐(4‐I‐Phe)‐Ala‐Ser‐Arg‐Lys(Dnp)‐amidation (MW: 1431.32 g/mol). It was obtained as a yellow solid. Yield: 48.0 mg, 0.034 mmol, 68%) UV purity (254 nm) = 97% (retention time = 3.63 min); LC‐MS m/z: [M + 2H]^2+^ calcd. for C_59_H_82_IN_17_O_17_, 714.66; found: 715.0.

#### 4.2.2 Peptide B

The amino acid sequence is Mca‐Glu‐Pro‐Leu‐Gly‐(4‐I‐Phe)‐Ala‐Ser‐Arg‐Lys(Dnp)‐amidation (MW: 1511.35 g/mol). It was obtained as a yellow solid. Yield: 41.3 mg, 0.028 mmol, 56%) UV purity (254 nm) = 97% (retention time = 6.30 min); LC‐MS m/z: [M + 2H]^2+^ calcd. for C_63_H_83_IN_16_O_20_, 756.18; found: 757.0.

### 4.3 Inhibitors

Marimastat (≥98% HPLC M2699‐5 mg), batimastat (≥98% HPLC, SML0041‐5 mg) and leupeptin (≥90% HPLC, L2884‐1 mg) were purchased from Sigma Aldrich/Merck (Merck KGaA, Darmstadt, Germany). Nafamostat mesylate (≥99% HPLC, HY‐B0190A‐5 mg) was purchased from MedChemExpress (MedChemExpress LLC, Monmouth Junction, USA). Ilomastat (≥90% HPLC, Cay14533‐5) was purchased from biomol (biomol GmbH, Hamburg, Germany). Inhibitors were prepared as 5 mM DMSO working stocks.

### 4.4 SVMP Assay

The SVMP activity was measured with substrate peptide **A**. The reactions consisted of 0.3 µg/100 µL venom (*C. atrox*, *B. jararaca*) or 6 µg/100 µL (*E. carinatus*) per reaction and a final concentration of 10 µM substrate **A** in a total volume of 100 µL. When inhibitors were tested, these were preincubated with the venom for 10 min at room temperature. Thereafter, data were collected on a Tecan Spark 10 M microplate reader at an excitation wavelength of 350 nm and emission wavelength of 445 nm at 25°C for 45 min. The linear slope of the first 20 min was calculated as the rate of reaction. As negative controls, the reaction mixtures were measured without venom. The inhibitors **1**–**3** were diluted in DMSO with a stock concentration of 5 mM and further diluted in assay buffer (150 mM NaCl, 50 mM Tris‐HCl pH 7.5) to final concentrations in a 3.16‐fold, semi‐logarithmic dilution series.

### 4.5 SVSP Assay

The SVSP assay buffer consists of 100 mM Tris‐HCl pH 8.5 with 100 mM NaCl. Reaction mixtures contain 2 µg/45 µL snake venom and a final substrate (Boc‐LRR‐AMC) concentration of 50 µM in a volume of 45 µL. When inhibitors were tested, these were preincubated with the venom for 10 min at room temperature. Thereafter, data were collected on a Tecan Spark 10 M microplate reader at an excitation wavelength of 380 nm and emission wavelength of 460 nm at 25°C for 45 min. The linear slope in the first 20 min was calculated as the rate of reaction. As negative controls, the reaction mixtures were measured without venom. The inhibitors **4** and **5** were diluted in DMSO with a stock concentration of 5 mM and further diluted in assay buffer to final concentrations in a 3.16‐fold, semi‐logarithmic dilution series.

### 4.6 Bloodserum Assay

For the bloodserum assay, fetal bovine bloodserum (F7524‐50 mL) and human serum (P2918‐20 mL) were purchased from Sigma Aldrich/Merck (Merck KGaA, Darmstadt, Germany). The human serum was filtered via a CHROMAFIL Xtra RC, 0.45 µm filter. The SVMP activity was measured with substrate peptide **B** and the SVSP activity with peptide Boc‐LRR‐AMC. The reactions were performed as described for the SVMP and SVSP assay, respectively. The peptide substrates were diluted from the stock solution to the final concentration in blood serum instead of assay buffer. Data of SVMP activity was collected on a Tecan Spark 10 M microplate reader at an excitation wavelength of 325 nm and emission wavelength of 420 nm at 25°C for 45 min; for SVSP excitation wavelength was 380 nm and emission wavelength 460 nm. IC_50_ measurements were performed as described above with the following venom concentrations: *C. atrox:* 0.3 µg/100 µL (SVMP, bovine); 1.0 µg/100 µL (SVMP, human); 2.0 µg/45 µL (SVSP, bovine); 6.0 µg/45 µL (SVSP, human). *B. jararaca:* 1 µg/100 µL (SVMP, bovine and human). *E. carinatus:* 6 µg/45 µL (SVSP, bovine and human).

All measurements for Michaelis–Menten kinetics, LoD and LoQ determination, and SVMP and SVSP inhibition were performed as triplicates. LoD and LoQ were determined according to the standard 3‐sigma and 10‐sigma approaches, respectively.

## Supporting Information

Additional supporting information can be found online in the Supporting Information section. SVMP/SVSP activity and inhibition assay data, chemical analytics, Figures S1–S10 and Table S1. **Supporting Fig. S1**: Determination of LoD and LoQ in Tris buffer. (A) In the SVMP assay setup for *B. jararaca* venom and (B) for *E. carinatus* venom. (C): In the SVSP assay with *B. jararaca* venom and (D) for *E. carinatus* venom. **Supporting Fig. S2**: Michaelis Menten kinetics to detect SVMP activity with peptide **A**. Venom of (A) *C. atrox*, (B) *B. jararaca*, (C) *E. carinatus*. **Supporting Fig. S3**: Michaelis Menten kinetics to detect SVSP activity with the peptide Boc‐LRR‐AMC. Venom of (A) *C. atrox*, (B) *B. jararaca*, (C) *E. carinatus.* **Supporting Fig. S4**: IC_50_ curves of metalloprotease inhibitors. (A) Batimastat with *C. atrox* venom. (B) Marimastat with *C. atrox* venom. (C) Ilomastat with *C. atrox* venom. (D) Batimastat with *B. jararaca* venom. (E) Marimastat with *B. jararaca* venom. (F) Ilomastat with *B. jararaca* venom. (G) Batimastat with *E. carinatus* venom. (H) Marimastat with *E. carinatus* venom and (I) Ilomastat with *E. carinatus* venom. **Supporting Fig. S5**: IC_50_ curves of serine protease inhibitors. (A) Nafamostat with *C. atrox* venom. (B) Nafamostat with *B. jararaca* venom. (C): Nafamoastat with *E. carinatus* venom. (D) Leupeptin with *C. atrox* venom. (E): Leupeptin with *B. jararaca* venom. (F) Leupeptin with *E. carinatus* venom. **Supporting Fig. S6**: Determination of LoD and LoQ in bovine and human bloodsera for SVMP activity. (A–D) in bovine serum: (A) *C. atrox* venom dose‐dependent time curve of SVMP activity. (B) LoD and LoQ determination with *C. atrox* venom. (B) Determination of LoD and LOQ with *B. jararaca* venom. (C) LoD and LoQ with *E. carinatus*. (E–G) in human serum: (E) LoD and LoQ determination with *C. atrox*. (F) LoD and LoQ with *B. jararaca* venom. (G) Determination of LoD and LoQ with *E. carinatus* venom. **Supporting Fig. S7**: Determination of LoD and LoQ in bovine and human bloodsera for SVSP activity. (A–D) in bovine serum: (A) *C. atrox* venom dose‐dependent time curve of SVSP activity. (B) LoD and LoQ determination with *C. atrox* venom. (B) Determination of LoD and LOQ with *B. jararaca* venom. (C) LoD and LoQ with *E. carinatus*. (E–G) in human serum: (E) LoD and LoQ determination with *C. atrox*. (F) LoD and LoQ with *B. jararaca* venom. (G) Determination of LoD and LoQ with *E. carinatus* venom. **Supporting Fig. S8**: IC_50_ curves of SVMP (A–D) and SVSP (E–H) inhibition in bovine and human sera. (A) *C. atrox* venom with marimastat in bovine serum. (B) *B. jararacas* venom with marimastat in bovine serum. (C) Marimastat with *C. atrox* venom in human serum. (D) *B. jararacas* venom with marimastat in human serum. (E) Nafamoastat with *C. atrox* venom in bovine serum. (F) *E. carinatus* venom with nafamostat in bovine serum. (G) Nafamostat with *C. atrox* venom in human serum. (H) Nafamostat with *E. carinatus* venom in human serum. **Supporting Fig. S9**: Mass spectrum and UV chromatogram (254 nm) of peptide **A**. **Supporting Fig. S10**: Mass spectrum and UV chromatogram (254 nm) of peptide **B. Supporting Table S1**: Viper venom composition for species under elucidation and exemplary proteases included in venom.

## Conflicts of Interest

The authors declare no conflicts of interest.

## Supporting information

Supplementary Material

## Data Availability

The data that support the findings of this study are available from the corresponding author upon reasonable request.
