# Supplementary material for: Snake Venom Protease Detection and Inhibition in Serum
Source: ChemMedChem. 2026 Mar 31;21(7):e202501099. doi: 10.1002/cmdc.202501099 (PMC13037691; doi:10.1002/cmdc.202501099)
Supplement: Supplementary file 1 — Supplementary Material [file CMDC-21-e202501099-s001.pdf]

Supporting Information

Snake Venom Protease Detection and Inhibition in Serum

Mareike Riedel[±], Christian Kersten[±,∇*]

[±]Institute of Pharmaceutical and Biomedical Science, Johannes Gutenberg University, Staudinger Weg 5, 55128 Mainz, Germany.

[∇] Institute for Quantitative and Computational Biosciences, Johannes Gutenberg-University, BioZentrum I, Hanns-Dieter-Hüsch Weg 15, 55128 Mainz, Germany

*Corresponding author:
E-mail address: kerstec@uni-mainz.de

Content:

SVMP and SVSP LoD and LoQ determination 2
Michaelis Menten kinetics 2
SVMP and SVSP inhibition 3
Bloodserum assay determining the SVMP and SVSP activity, LoD and LoQ 4
Inhibition of SVMP and SVSP activity in bloodserum 5
LC/MS analytics 6
Viper venom SVMP and SVSP components 7

SVMP and SVSP LoD and LoQ determination

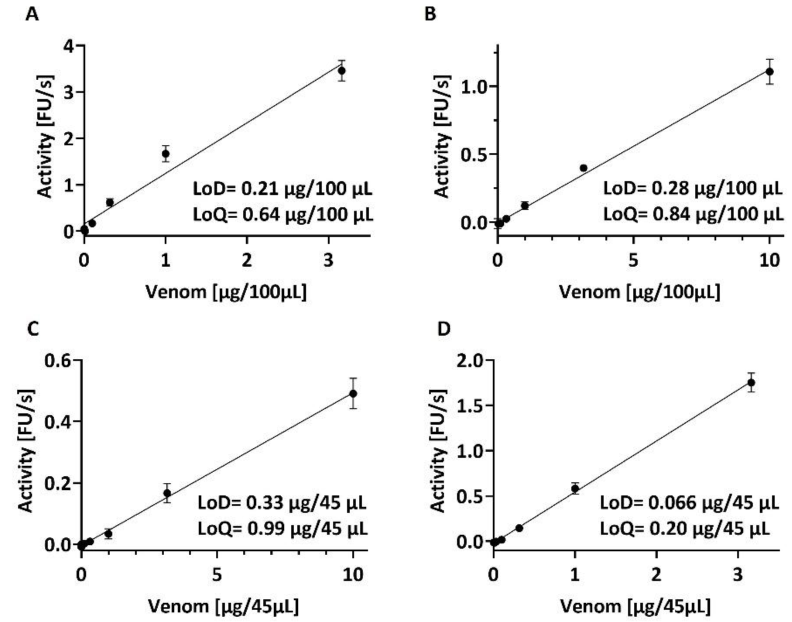

Figure S 1. Determination of LoD and LoQ in Tris buffer. **A:** In the SVMP assay setup for *B. jararaca* venom and **B:** for *E. carinatus* venom. **C:** In the SVSP assay with *B. jararaca* venom and **D:** for *E. carinatus* venom.

Michaelis Menten kinetics

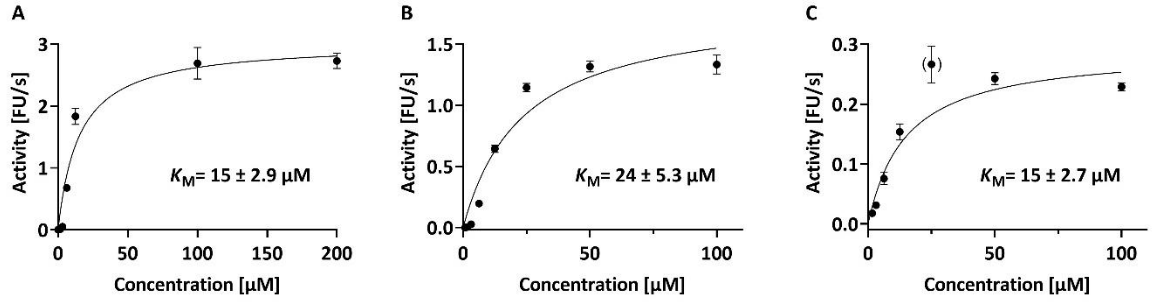

Figure S 2: Michaelis Menten kinetics to detect SVMP activity with peptide **A**. Venom of **A:** *C. atrox*, **B:** *B. jararaca*, **C:** *E. carinatus*.

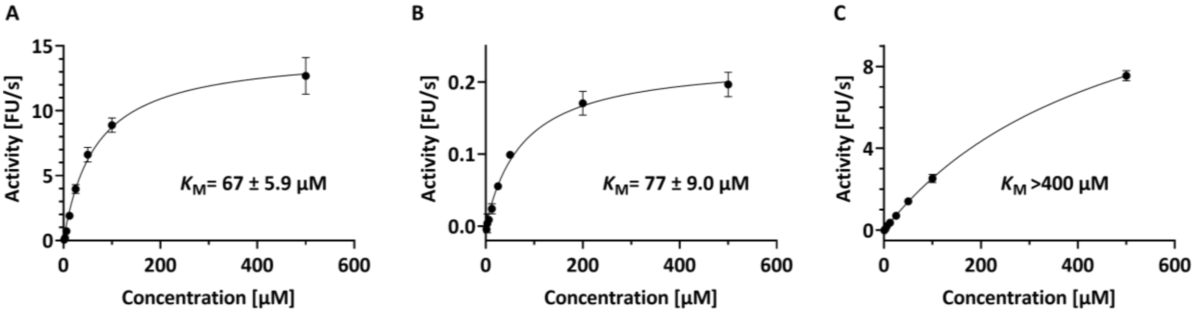

Figure S 3: Michaelis Menten kinetics to detect SVSP activity with the peptide Boc-LRR-AMC. Venom of **A:** *C. atrox*, **B:** *B. jararaca*, **C**: *E. carinatus*.

SVMP and SVSP inhibition

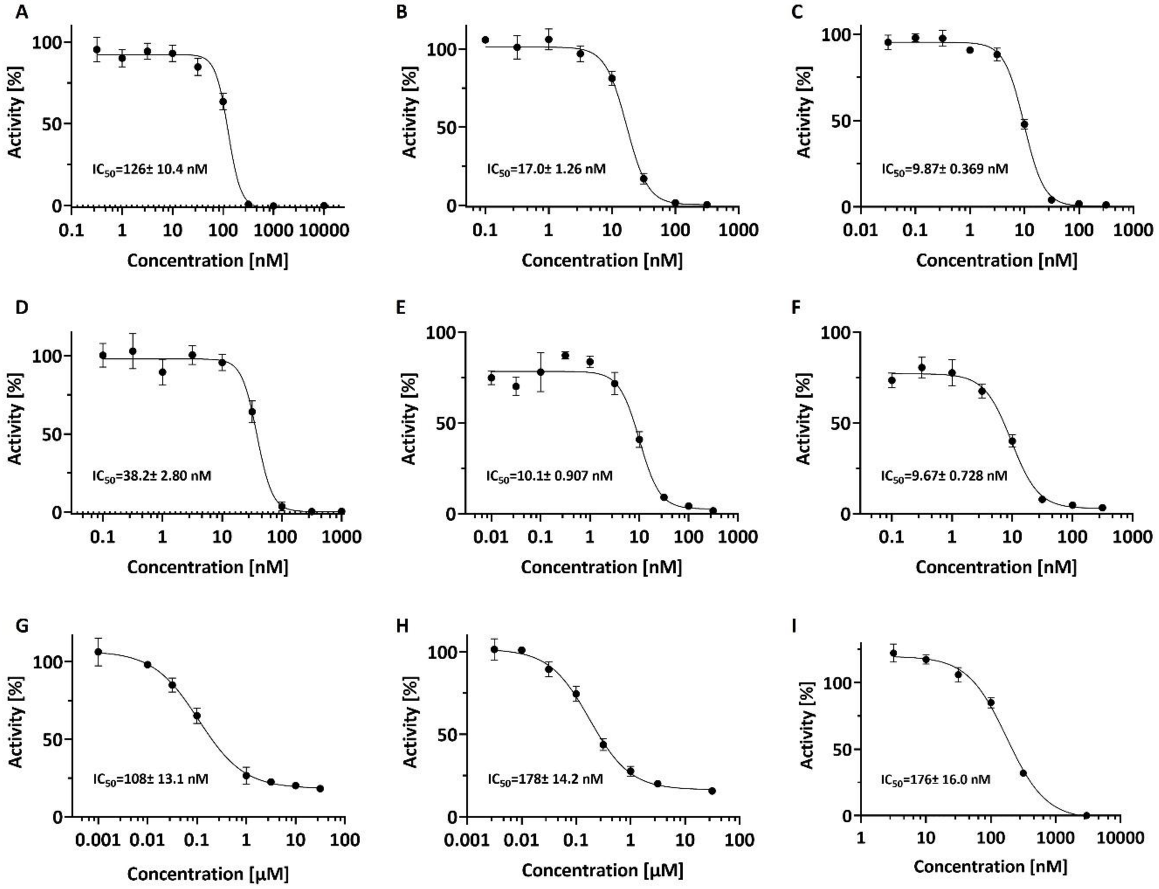

Figure S 4. IC_{50} curves of metalloprotease inhibitors. **A:** Batimastat with *C. atrox* venom**. B:** Marimastat with *C. atrox* venom. **C:** Ilomastat with *C. atrox* venom. **D:** Batimastat with *B. jararaca* venom. **E:** Marimastat with *B. jararaca* venom. **F:** Ilomastat with *B. jararaca* venom. **G:** Batimastat with *E. carinatus* venom. **H:** Marimastat with *E. carinatus* venom and **I:** Ilomastat with *E. carinatus* venom.

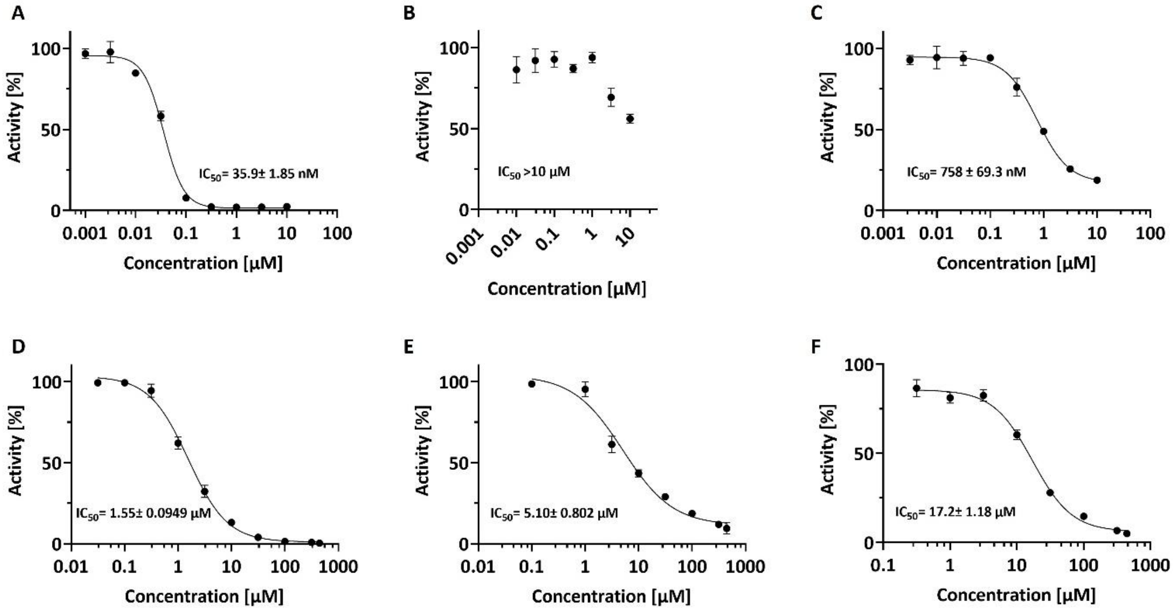

Figure S 5. IC_{50} curves of serine protease inhibitors. **A:** Nafamostat with *C. atrox* venom**. B:** Nafamostat with *B. jararaca* venom. **C:** Nafamoastat with *E. carinatus* venom. **D:** Leupeptin with *C. atrox* venom. **E:** Leupeptin with *B. jararaca* venom. **F:** Leupeptin with *E. carinatus* venom.

Blood serum assay determining the SVMP and SVSP activity, LoD and LoQ

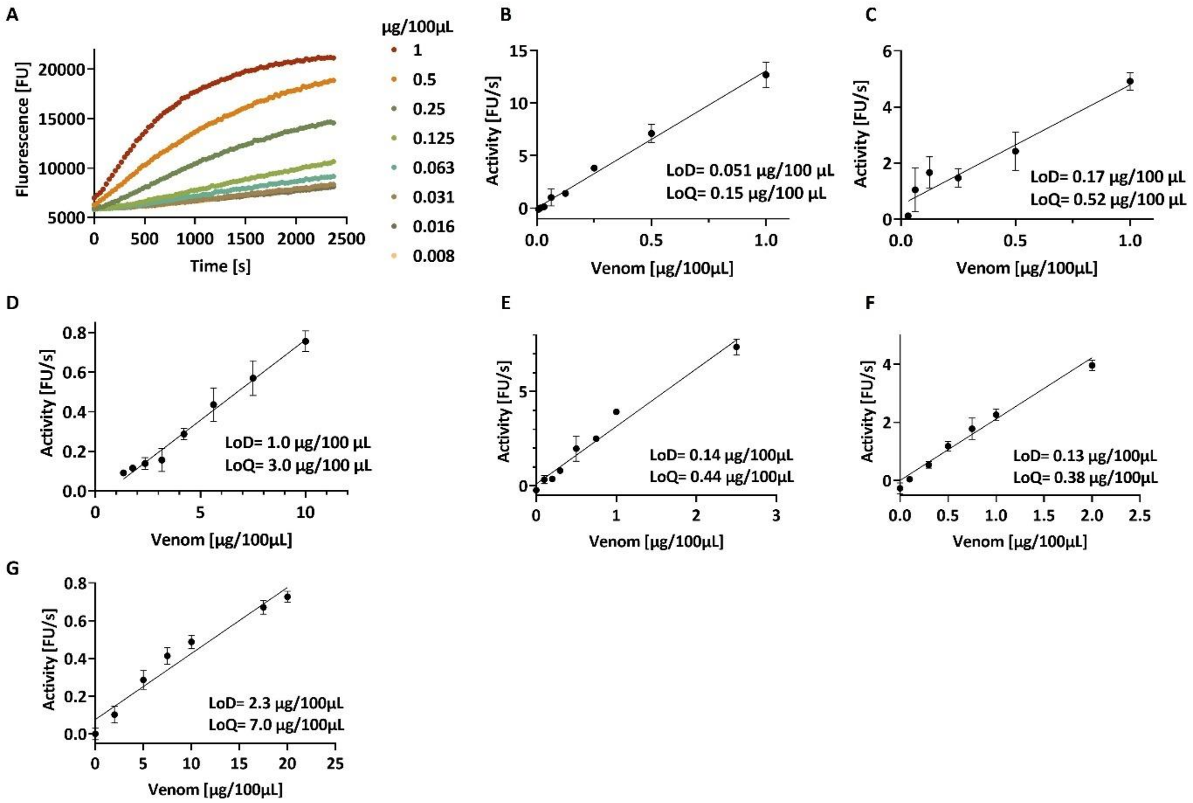

Figure S 6. Determination of LoD and LoQ in bovine and human bloodsera for SVMP activity. **A-D** in bovine serum: **A**: *C. atrox* venom dose-dependent time curve of SVMP activity. **B:** LoD and LoQ determination with *C. atrox* venom. **B:** Determination of LoD and LOQ with *B. jararaca* venom. **C:** LoD and LoQ with *E. carinatus*. **E-G** in human serum: **E:** LoD and LoQ determination with *C. atrox*. **F:** LoD and LoQ with *B. jararaca* venom. **G:** Determination of LoD and LoQ with *E. carinatus* venom.

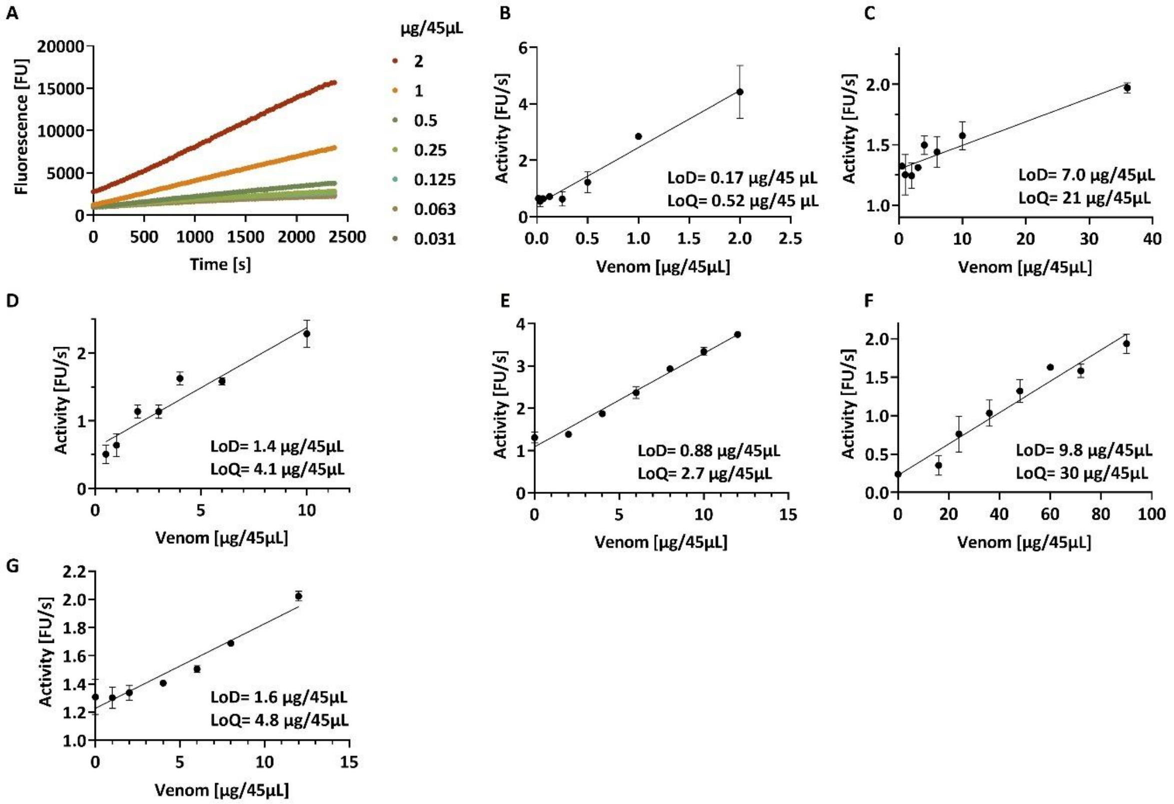

Figure S 7**:** Determination of LoD and LoQ in bovine and human bloodsera for SVSP activity. **A-D** in bovine serum: **A**: *C. atrox* venom dose-dependent time curve of SVSP activity. **B:** LoD and LoQ determination with *C. atrox* venom. **B:** Determination of LoD and LOQ with *B. jararaca* venom. **C:** LoD and LoQ with *E. carinatus*. **E-G** in human serum: **E:** LoD and LoQ determination with *C. atrox*. **F:** LoD and LoQ with *B. jararaca* venom. **G:** Determination of LoD and LoQ with *E. carinatus* venom.

Inhibition of SVMP and SVSP activity in blood serum

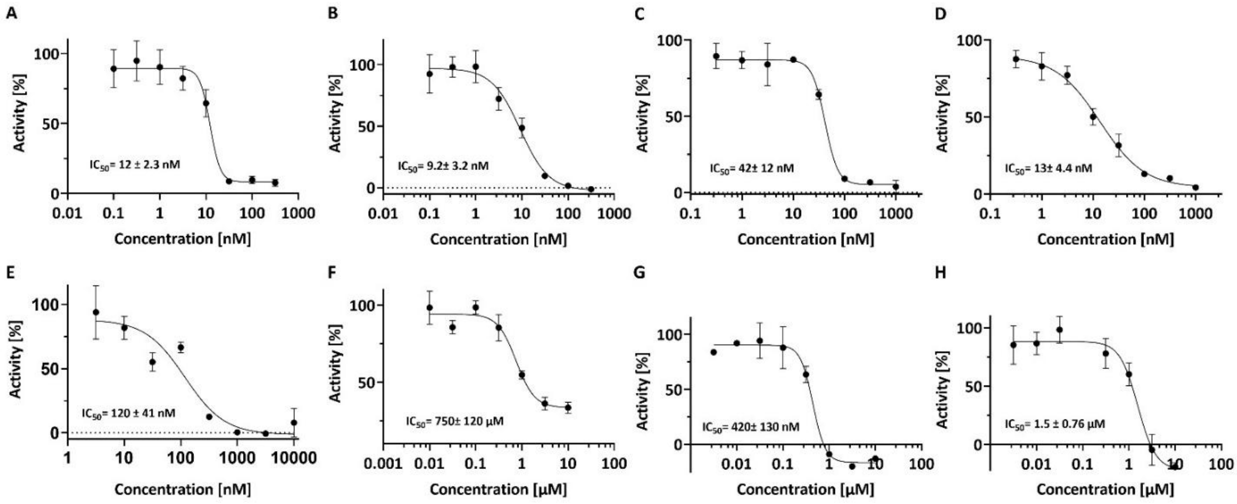

Figure S 8: IC_{50} curves of SVMP (**A**-**D**) and SVSP (**E**-**H**) inhibition in bovine and human sera. **A:** *C. atrox* venom with marimastat in bovine serum. **B:** *B. jararacas* venom with marimastat in bovine serum. **C:** Marimastat with *C. atrox* venom in human serum. **D:** *B. jararacas* venom with marimastat in human serum. **E:** Nafamoastat with *C. atrox* venom in bovine serum. **F:** *E. carinatus* venom with nafamostat in bovine serum. **G:** Nafamostat with *C. atrox* venom in human serum. **H:** Nafamostat with *E. carinatus* venom in human serum.

LC/MS analytics

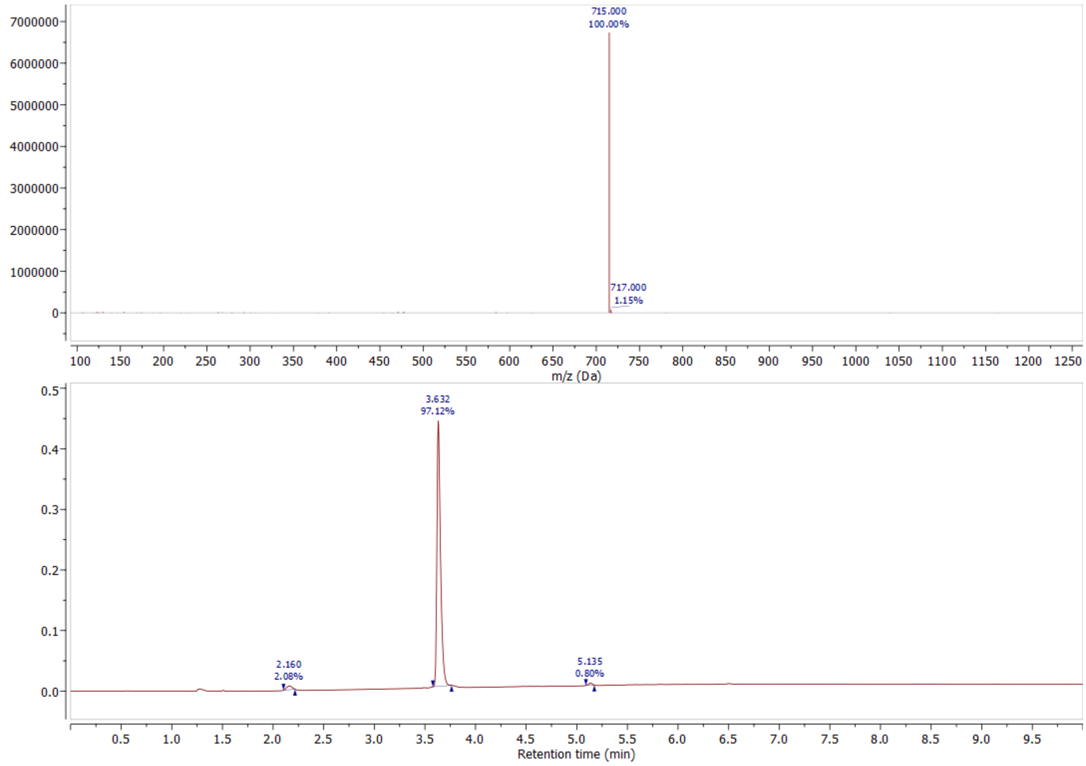

Figure S 9: Mass spectrum and UV chromatogram (254 nm) of peptide **A.**

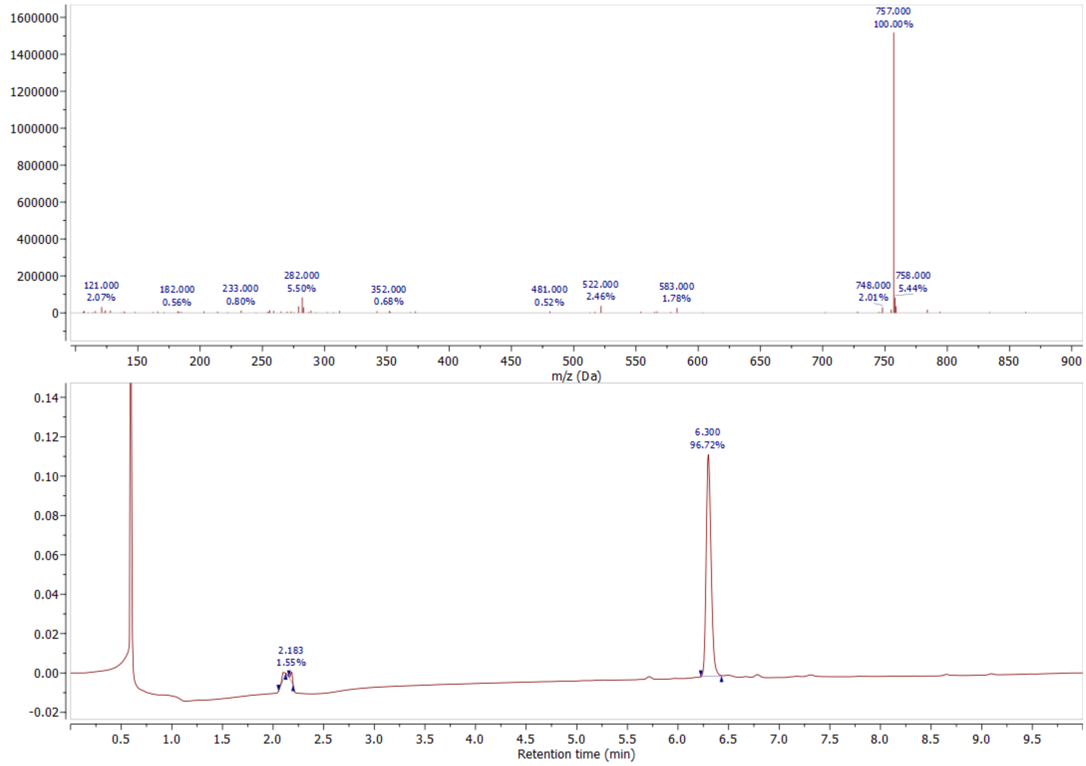

Figure S 10: Mass spectrum and UV chromatogram (254 nm) of peptide **B.**

Viper venom SVMP and SVSP components

Table S 1: Viper venom composition for species under elucidation and exemplary proteases included in venom.

Venom	protease type (% of venom content) [3]	reported proteases included	Uniprot-ID
C. atrox	SVMP (49.7%)	Zinc metalloproteinase-disintegrin-like VAP2B	Q90282
		Zinc metalloproteinase-disintegrin-like VAP1	Q9DGB9
		Snake venom metalloproteinase atrolysin-D	P15167
	SVSP (19.8 %)	Snake venom serine protease catroxase-1	Q8QHK3
		Snake venom serine protease catroxase-2	Q8QHK2
B. jararaca	SVMP (35.6 %)	Zinc metalloproteinase-disintegrin-like bothropasin	O93523
		Zinc metalloproteinase/disintegrin	Q0NZX5
	SVSP (13.7 %)	Thrombin-like enzyme bothrombin	P81661
		Thrombin-like enzyme KN-BJ 2	O13069
E. carinatus	SVMP (32.8 %)	Zinc metalloproteinase-disintegrin-like ecarin	Q90495
	SVSP (4.8 %)	Serine protease (not further specified)	E9JG22
		Serine protease (not further specified)	E9JG24
